# The effect of prophylactic chemotherapy on treatment outcome of postmolar gestational trophoblastic neoplasia

**DOI:** 10.1186/s12905-022-02134-w

**Published:** 2023-01-02

**Authors:** Yuanyuan Liu, Yaqiong Ye, Xiaodong Cheng, Weiguo Lu, Xing Xie, Xinyu Wang, Xiao Li

**Affiliations:** 1 Department of Gynecologic Oncology, Women’s Hospital, Zhejiang University School of Medicine, No. 1 Xueshi Road, Hangzhou, 310006 Zhejiang China; 2 Zhejiang Provincial Key Laboratory of Precision Diagnosis and Therapy for Major Gynecological Diseases, Women’s Hospital, Zhejiang University School of Medicine, Hangzhou, Zhejiang China; 3 Cancer Research Institute of Zhejiang University, Hangzhou, Zhejiang China; 4 Ninghai Second People’s Hospital, Ninghai, 315600 Zhejiang China; 5 Center for Uterine Cancer Diagnosis and Therapy Research of Zhejiang Province, Hangzhou, 310006 Zhejiang China

**Keywords:** Hydatidiform mole, Prophylactic chemotherapy, Gestational trophoblastic neoplasia, Chemotherapy resistance

## Abstract

**Objective:**

To evaluate whether prophylactic chemotherapy (P-chem) increased the drug resistance rate of postmolar GTN and whether the first-line chemotherapy should be different from P-chem.

**Methods:**

Postmolar GTN received P-Chem was defined as P-Chem group. Postmolar GTN without P-chem was randomly selected as control group according to the ratio of 1:3 (P-chem:control) and matched by age for low risk and high risk GTN separately.

**Results:**

Totally 455 low-risk and 32 high-risk postmolar GTN patients were included. WHO risk score, chemotherapy cycles to achieve hCG normalization and resistant rate were similar between P-chem (27 cases) and control (81 cases) group. Among low-risk GTN patients, interval from hydatidiform mole to GTN was significantly longer in P-chem group than control (44 vs 69 days, P = 0.001). Total chemotherapy cycles and resistant rate were similar between low-risk GTN treated with same agent as P-chem (group A) and alternative agent (group B). But group A needed more chemotherapy cycles to achieve hCG normalization than group B.

**Conclusions:**

P-chem delayed the time to GTN diagnosis, but didn’t increase risk score or lead to drug resistance of postmolar GTN. Alternative agent different from P-chem had the potential of enhancing chemotherapy response in low- risk postmolar GTN.

**Supplementary Information:**

The online version contains supplementary material available at 10.1186/s12905-022-02134-w.

## Background

Gestational trophoblastic disease (GTD) is a group of uncommon conditions associated with pregnancy [1]. Among them, hydatidiform mole (HM) is benign and the most common form of GTD. HM consists of complete hydatidiform mole (CHM) and partial hydatidiform mole (PHM). About 15–29% of CHM and 0.5–6% PHM might progress to malignant gestational trophoblastic neoplasia (GTN) and require further treatment [2–6]. High risk factors for malignant transformation of HM include: age > 40 years, preevacuation human chorionic gonadotropin (hCG) levels > 100,000 IU/L, enlarged uterus for gestational age and/or theca lutein cysts larger than 6 cm [7]. For high-risk HM, the rate of malignant transformation was reported from 30 to 50%, which was much higher than that of HM without high risk factors [8–10].

The use of prophylactic chemotherapy (P-chem) was considered as an effective method to prevent malignant transformation of high-risk HM, after it was first introduced in 1966 [11]. An updated Cochrane Review in 2017 concluded that P-chem might reduce the risk of post-molar GTN to 3–8% in women with CHM who are at high risk of malignant transformation [12]. However, P-chem cannot be recommended routinely for high risk HM nowadays, since it has been reported that P-chem might exert adverse effects on subsequent follow-up and treatment of postmolar GTN [12–15]. As previous studies stated, P-chem might delay the time to diagnosis of GTN and increase the risk of subsequent drug resistance, but these effects were very uncertain due to the small samples and limited evidences [9, 15, 16]. Nevertheless, the International Federation of Gynecology and Obstetrics (FIGO) and National Comprehensive Cancer Network (NCCN) still stated that P-Chem could be administered for HM under certain circumstances, in which the risk of postmolar GTN is much greater than normal or where reliable follow-up is not possible [1, 7].

It was reported that women of lower socioeconomic status had a tenfold higher risk of developing hydatidiform mole than their wealthier counterparts [17, 18]. For these patients, even basic health care was difficult to obtain. Thus, in economically underdeveloped areas, clinicians would incline to treat high-risk HM patients with P-chem, and P-chem was even to be adopted as routine clinical practice in some countries [19]. HM was more common in China and some other developing countries, with the incidence of 2 per 1000 pregnancies, which was much higher than the European and American countries [20]. Even in developed countries, follow-up compliance among HM patients after evacuation was poor. Only 7% to 36% of low-income patients were fully in accordance with doctor’s suggestion in United States [21, 22]. This phenomenon was also common in other developed countries such as South Korea, Japan and Netherlands [15, 23, 24]. Thus, P-Chem might still be used under certain situations in both developing and developed countries.

Due to the rare conditions and insufficient evidences, it remains unclear whether P-chem delays the time of effective treatment or increases drug resistance of postmolar GTN. In order to provide solid evidences for clinical practice of GTD, disease status, drug resistant rate and subsequent optimal management for postmolar GTN received P-chem are needed for further study. In present retrospective study, we try to evaluate the effect of P-chem on drug sensitivity of subsequent postmolar GTN and to determine whether an alternative agent different from P-chem should be prescribed as first-line chemotherapy for reducing resistance to initial treatment.

## Materials and methods

### Study design

We retrospectively reviewed the records of postmolar GTN patients managed at Women's hospital, Zhejiang University School of Medicine between January 1, 2008 and December 31, 2017. Postmolar GTN that previously received P-Chem and met following criteria was defined as P-Chem group: (1) Molar pregnancy was confirmed by pathologic diagnosis. (2) P-chem was performed within one week after evacuation. (3) Single drug chemotherapy was used for P-chem, including MTX, 5-FU or Act-D. (4) The criteria used to indicate P-chem include: HM patients with high risk factors for malignant transformation or HM patients who cannot be reliably followed up. For control group, patients were randomly selected from postmolar GTN without P-chem according to the ratio of 1:3 (P-chem:control) matching by age for high and low risk GTN separately.

Postmolar GTN was reevaluated based on the 2018 FIGO criteria [1]: (1) the plateau of hCG lasts for four measurements over a period of 3 weeks or longer; (2) a rise in hCG for three consecutive weekly measurements over at least a period of 2 weeks or more; (3) a histological diagnosis of choriocarcinoma; (4) residual HM or pregnancy is excluded. P-chem was prescribed within 1 week after evacuation in present study. The risk score was calculated based on the modified World Health Organization (WHO) scoring system [25].

According to the regulations of our hospital, all GTN patients were evaluated by pelvic ultrasound, chest X-ray (CXR) and serum hCG. The patients with pulmonary metastases detected by CXR would receive abdomen and brain CT or MRI, while the patients with negative CXR would receive chest CT for staging. Serum hCG normalization was defined as < 5.3 IU/L. The chemotherapy regimen for GTN was prescribed according to FIGO guideline. During treatment, serum hCG levels were monitored weekly. Drug failure was defined as serum hCG levels not showing a logarithmical decline after two cycles, and elevated or roughly same hCG level or newly developed metastasis after one cycle. This study was reviewed and approved by the Ethical Committee of Women's hospital, Zhejiang University School of Medicine (20190074). All the study procedures were carried out in accordance with the principles of the Declaration of Helsinki. Because of the retrospective character of the study, informed consent was waived by Ethical Committee of Women's hospital, Zhejiang University School of Medicine.

### Variables

The clinical information of each selected patient was collected. For HM stage, the variables included age, gravidity, parity, gestational age, histology of HM, hCG level before evacuation (IU/L), diameter of theca lutein cyst, uterine size (cm), history of HM in previous gestation, regimen of P-chem. For GTN stage, the variables included interval from HM to GTN (days), hCG level before first-line chemotherapy (IU/L), largest dimension of tumor (cm), number and location of metastases, previous failed chemotherapy, FIGO stage, chemotherapy regimen, chemotherapy cycles to achieve hCG normalization and drug response.

### Statistical analysis

SPSS 25.0 statistical software was used for statistical analyses. Mann–Whitney test was used for continuous variables, and Chi-square test or Fisher's exact test was used for categorical variables. For all analyses, an alpha level < 0.05 was considered statistically significant.

## Results

### The sociodemographic data of HM in control and P-chem group

Totally 455 low-risk and 32 high-risk postmolar GTN patients were treated at our GTD center between January 1, 2008 and December 31, 2017. In P-chem group, 24 cases were low-risk GTN and 3 cases were high-risk GTN. In control group, 72 low-risk GTN and 9 high-risk GTN without previous P-chem were randomly selected. The rate of high-risk GTN in 27 patients who received P-chem was higher than that in 460 postmolar GTN who didn’t receive P-chem (11.1% vs 6.3%, P = 0.327), although the difference was no significant. These results suggested that P-chem might have the potency to increase the severity of postmolar GTN.

The sociodemographic data of HM in both groups were presented in Table 1. No significant difference was observed in all variables between P-chem group and control. For low-risk GTN, further analysis either revealed no significant difference in all variables of HM except gravidity (Additional file 1: Table S1). For high-risk GTN, no significant difference was observed in age, gestational history, gestational age and hCG before evacuation between both groups, while other variables were not compared due to data missing (Additional file 1: Table S2).

**Table 1:** The sociodemographic data of hydatidiform mole in control and P-chem group

Variables*	Control (n = 81)	P-chem (n = 27)	*P* value
Age (years)	29 (18–54)	29 (20–53)	0.837
Gravidity	2 (1–7)	2 (1–7)	0.142
Parity	1 (0–3)	0 (0–2)	0.797
Gestational age(weeks)	9.3 (6.3–25.1)	9.5 (4.7–21.0)	0.932
Histology of HM			
Complete HM	75 (93%)	24 (89%)	0.688
Partial HM	6 (7%)	3 (11%)	
hCG before evacuation (IU/L)	225,000 (60,000–225,000)	200,000 (1000–1,278,808)	0.635
Diameter of theca lutein cyst			
≤ 6 cm	27 (33%)	5 (18%)	0.535
> 6 cm	3 (4%)	1 (4%)	
Missing data	51 (63%)	21 (78%)	
Excessive uterine enlargement			
No	22 (27%)	2 (7%)	0.063
Yes	7 (9%)	4 (15%)	
Missing data	52 (64%)	21 (78%)	
History of HM in previous gestation			
No	81 (100%)	26 (96%)	0.250
Yes	0 (0%)	1 (4%)	

*P-chem* prophylactic chemotherapy, *HM* hydatidiform mole

*Continuous variables are reported as medians(range), and categorical variables are reported as raw numbers with proportions

### The effect of P-chem on drug response of low-risk postmolar GTN

The clinical characteristics of low-risk postmolar GTN were evaluated in Table 2. Interval from HM to GTN was significantly longer in P-chem group than control (44 vs 69 days, P = 0.001), while the serum hCG level before first-line chemotherapy was significantly lower in P-chem group than control (4503.5 vs 415.8 IU/L, p = 0.000). These data indicated that P-chem might postpone the diagnosis time of postmolar GTN but decrease the hCG level at malignant transformation. In a result, there was no significant difference for FIGO score between both groups.

**Table 2:** The clinical characteristics of low-risk postmolar GTN

Variables*	Control (n = 72)	P-chem (n = 24)	*P* value
Interval from HM to GTN (days)	44 (11–2555)	69 (14–300)	0.001
Largest dimension of tumor (cm)			
< 3	40 (56%)	4 (17%)	0.181
≥ 3	23 (32%)	6 (25%)	
Number of metastases	0 (0–9)	0 (0–9)	0.054
hCG before first-line chemotherapy (IU/L)	4503.5 (5.3–514,972.0)	415.8 (10.7–44,808.0)	0.000
FIGO stage			
I	22 (31%)	11 (46%)	0.414
II	1 (1%)	0 (0%)	
III	49 (68%)	13 (54%)	
WHO risk score	2 (0–6)	2 (0–6)	0.208
First-line chemotherapy agent			
Methotrexate	69 (96%)	13 (54%)	0.000
Actinomycin D	3 (4%)	8 (33%)	
5-Fluorouracil	0 (0%)	3 (13%)	
Resistant to first-line drug	29 (40%)	10 (42%)	0.904
Resistant to first-line MTX	26 (38%)	5 (38%)	0.066
Resistant to second-line drug	6 (21%)	2 (20%)	1.000
Resistant to single-agent drug	8 (11%)	4 (17%)	0.487
Chemotherapy cycles to hCG normalization	4 (1–9)	3 (1–7)	0.157
Total chemotherapy cycles	6 (1–12)	6 (3–11)	0.508

*P-chem* prophylactic chemotherapy, *HM* hydatidiform mole, *GTN* gestational trophoblastic neoplasia

*Continuous variables are reported as medians(range), and categorical variables are reported as raw numbers with proportions

Methotrexate (MTX) was prescribed as first-line chemotherapy to 96% patients in control group and to 54% patients in P-chem group. The failure rate of first-line or second-line chemotherapy was not significantly different between both groups. Due to the different first-line drug used in control and P-chem group, we further compared the resistant rate of single-agent chemotherapy and found there was no significant difference between both groups. Moreover, the chemotherapy cycles to hCG normalization and total chemotherapy cycles were similar in both groups (Table 2). These results indicated that P-chem didn’t increase drug resistance of low-risk postmolar GTN.

### The effect of alternative agent as first-line chemotherapy on drug response of low-risk GTN in P-chem group

The detailed chemotherapy regimens used for low-risk GTN in P-chem group were listed in Table 3. The resistant rate of first-line chemotherapy was 38.5% for MTX, 37.5% for actinomycin D (Act-D) and 66.7% for 5-fluorouracil (5-FU). These patients were further divided into two subgroups. Group A included 11 patients who received first-line agent as same as P-chem, while group B included 13 patients whose first-line treatments were changed to another single-agent chemotherapy (Table 4). There were no significant differences for age, gravidity, parity, hCG level before evacuation, interval from HM to GTN, and FIGO score between group A and B. Unexpectedly, total chemotherapy cycles, resistant rate of first-line chemotherapy and resistant rate of single-agent chemotherapy were similar between two groups. Howerver, the patients in group A needed more chemotherapy cycles to achieve hCG normalization than group B (4.4 vs 2.8, p = 0.024). Even in 22 patients received MTX for P-chem, failure rate of MTX as first-line chemotherapy was similar to that of alternative agent (45.5% vs 45.5%, p = 1.000). However, the number of courses to achieve hCG normalization in the MTX group was 1 more course than that in the alternative agent group (3 vs 2, p = 0.004) (Additional file 1: Table S3). These results indicated that alternative agent might have the potential of increasing drug response of first-line treatment in low-risk postmolar GTN.

**Table 3:** The detailed chemotherapy regimen used for low-risk GTN in P-chem group

P-chem (n)	First-line drug (n)	Resistant rate (n, %)	Second-line drug (n)	Resistant rate (n, %)
MTX (22)	MTX (11)	5 (45.5)	Act-D (5)	1 (20.0)
	Act-D (8)	3 (37.5)	EMA-CO (3)	1 (33.3)
	5-FU (3)	2 (66.7)	Act-D (2)	0
Act-D (1)	MTX (1)	0		
5-FU (1)	MTX (1)	0		

*P-chem* prophylactic chemotherapy, *MTX* methotrexate, *Act-D* actinomycin D, *5-FU* 5-fluorouracil, *EMA-CO* etoposide, methotrexate, actinomycin D, cyclophosphamide, vincristine

**Table 4:** The effect of alternative agent as first-line chemotherapy on treatment outcome of low-risk GTN

Variables*	Group A (n = 11)	Group B (n = 13)	*P* value
Age (years)	25 (21–49)	29 (22–53)	0.190
Gravidity	3 (1–5)	3 (1–7)	0.700
Parity	0 (0–2)	1 (0–1)	0.176
hCG before evacuation (IU/L)	200,000 (1000–1,278,808)	202,418 (20,000–998,083)	0.620
Interval from HM to GTN (days)	62 (14–153)	100 (44–300)	0.082
Largest dimension of tumor (cm)	1.6 (0–7.3)	0.6 (0–3.2)	0.089
Number of metastases	0 (0–2)	0 (0–9)	0.540
hCG before first-line chemotherapy (IU/L)	129 (11–44,808)	530 (13–15,885)	0.794
FIGO stage			
I	2 (18%)	9 (69%)	0.012
II	0 (0%)	0 (0%)	
III	9 (82%)	4 (31%)	
WHO risk score	2 (0–4)	1 (0–6)	0.412
Resistant to first-line drug	5 (45%)	5 (38%)	0.729
Resistant to single-agent drug	1 (9%)	3 (23%)	0.596
Chemotherapy cycles to hCG normalization	4.4 ± 1.7	2.8 ± 1.5	0.024
Total chemotherapy cycles	6 (5–11)	6 (3–9)	0.261

*HM* hydatidiform mole, *GTN* gestational trophoblastic neoplasia

*Continuous variables are reported as medians (range) or mean ± SD, and categorical variables are reported as raw numbers with proportions

### The clinical characteristics of high-risk postmolar GTN in control and P-chem group

Among high-risk postmolar GTN, there were no significant differences for interval from HM to GTN, largest dimension of tumor, number of metastases, hCG before first-line chemotherapy and FIGO score between control and P-chem group (Table 5). All patients received EMA-CO (etoposide, methotrexate, actinomycin D, cyclophosphamide, vincristine) as first-line chemotherapy. The resistant rate of first-line chemotherapy, chemotherapy cycles to hCG normalization and total chemotherapy cycles had no significant difference between control and P-chem group.

**Table 5:** The clinical characteristics and chemotherapy response of high-risk postmolar GTN

Variables*	Control (n = 9)	P-chem (n = 3)	*P* value
Interval from HM to GTN (days)	62 (27–1095)	87 (54–2920)	0.309
Largest dimension of tumor(cm)	5.0 (1.1–9.5)	5.5 (3.2–9.7)	0.459
Number of metastases	2 (0–11)	1 (0–5)	0.509
hCG before first-line chemotherapy(IU/L)	92,561 (11,235–1,000,000)	22,913 (17,625–126,575)	0.405
FIGO stage^a^			
I	0 (0%)	1 (33%)	
II	1 (11%)	0 (0%)	
III	6 (67%)	2 (67%)	
IV	2 (22%)	0 (0%)	
WHO risk score	7 (7–14)	9 (7–10)	0.426
Resistant to first-line drug	1 (11%)	0/3 (0%)	1.000
Chemotherapy cycles to hCG normalization	4 (3–8)	4 (4–4)	0.352
Total chemotherapy cycles	8 (6–9)	7 (7–10)	0.924

*P-chem* prophylactic chemotherapy, *HM* hydatidiform mole

^a^Variables were not compared due to small data

*Continuous variables are reported as medians (range), and categorical variables are reported as raw numbers with proportions

## Discussion

It is non-controversial that P-chem is not routinely recommended in nowadays [3, 13, 26]. But as FIGO cancer report and NCCN guideline stated, P-chem could be used under special situations and it was effective especially developing countries [1, 7]. We found that the proportion of high-risk GTN in 27 patients who received P-chem was higher than that in 460 postmolar GTN who didn’t receive P-chem (11.1% vs 6.3%), although the difference was not significant, which also supported that P-chem should be administered in cautious. Up to date, no optimal regimen for P-chem has been defined. Usually, MTX or Act-D is suggested for P-chem [7]. But in china, 5-FU is also prescribed as P-chem agent based on the theory that chemotherapy agents that have known activity against GTN may prevent progression to GTN [12]. The course of chemotherapy was not clearly defined in previous study and varied in our study. Most studies administered one course [8, 10, 27, 28], while Fasoli administered three courses of MTX [29]. The regimen and timing for P-chem were also varied, including 5-day Act-D, 5-day or 8-day MTX and single-dose Act-D [8–10, 15, 28–32]. Although most studies administered P-chem before or during evacuation of HM [8, 10, 28], it carried the inherent risk of over-treating for those patients with histological confirmed non-molar hydropic abortions [33]. In present study, P-chem was performed within 1 week after evacuation, which is similar with previous studies that administered P-chem within 1 week or 3 weeks after evacuation [9, 20, 34].

The interval from pervious HM to GTN is one component in WHO scoring system since 2000 [25]. In our study, the diagnosis of GTN was made approximately 25 days later in the P-Chem group (including high-risk and low-risk GTN) than control, which is consistent with previous report [15]. But hCG level in P-Chem group was lower than control, which might offset the role of delayed diagnosis. In a result, WHO risk score was similar in both groups, which might further determine the treatment outcome of postmolar GTN.

Due to limited contributing data, drug resistance following P-chem was inadequately evaluated in previous study [12]. Kim et al. deduced that P-chem might increase resistance to subsequent chemotherapy for GTN, because they found that P-chem group needed more chemotherapy courses than control (2.5 vs 1.4, p < 0.005) [15]. However, their data suggested that both 4 GTN in P-chem group and 10 GTN in control achieved complete remission following initial treatment for GTN. Due to the small sample in Kim’s study, the evidence is inadequate to draw a definite conclusion. On the contrary, Uberti et al. analyzed 265 patients with high-risk HM, and found that P-chem didn’t increase resistant rate or chemotherapy courses of postmolar GTN [10]. Consistent with Uberti’s reports, our results also found that P-Chem didn’t increase chemotherapy cycles and resistant rate of chemotherapy in both low risk and high risk GTN. Due to the different first-line agent used in low risk GTN, resistant rate of single-agent chemotherapy was further compared and no difference was revealed. Combined previous studies with our data, P-chem is not recommended because the similar therapeutic effects were obtained in both group and even at least one course of single agent would give adverse effect to patients who might not develop to GTN without P-chem [3, 13, 35]. But, for patients who had already received P-chem, our results also indicated that P-chem don’t contribute to drug resistance for postomar GTN treatment.

As we know, the drugs used for P-Chem are also used for GTN treatment, so it is assumed that an alternative agent could be used for following low-risk GTN in order to prevent resistance to first-line chemotherapy [13]. But, Uberti’s results revealed that alternative agent did not reduce resistant rate, after we further calculated their raw data (25.0% vs 33.3%, p = 0.645) [10]. Similar with Uberti’s study, our results found that alternative agent as first-line chemotherapy did not reduce the resistant rate of low-risk postmoalr GTN. Moreover, resistant rate of single-agent chemotherapy and total chemotherapy cycles in patients treated by alternative agent were also similar with that treated by agent same as P-chem, even among 22 patients who were administered MTX as P-chem. But interestingly, further statistics revealed that alternative agent reduced about 1.6 chemotherapy cycles to achieve hCG normalization when 24 low-risk GTN were included, and drugs different from MTX reduced 1 chemotherapy cycle when only 22 patients received MTX for P-chem were included. These results indicated that alternative agent had the potential of increasing chemotherapy response of low-risk GTN, despite that the drug resistant rate didn't change.

Present study focuses on the effect of P-chem and alternative agent on the chemotherapy response of subsequent postmolar GTN, based on a relatively large number of cases. However, our study was conducted in single GTD center and the data was collected retrospectively. Due to the retrospective nature of this study, we do not have complete follow-up information from the onset of HM, such as the number of patients developed post-molar GTN. Small sample size and varied chemotherapy regimens of present study contributed to the relatively weak statistical power of analysis. Thus, our results should be interpreted with caution, and further prospective randomized control trials are recommended to provide sufficient evidences about the use of P-Chem in clinical practice.

## Conclusion

In summary, we found that P-chem delayed the time to GTN diagnosis, but did not increase risk score or lead to subsequent drug resistance of postmolar GTN. But, P-Chem should be adopted in caution since it might have the potency to increase the severity of postmolar GTN. Alternative agent different from P-chem has the potential of increasing chemotherapy response in low-risk postmolar GTN. Our study investigated the effect of P-chem on chemo-sensitivity of subsequent postmolar GTN, which will provide clinical evidences for future practice.

## Supplementary Information

**Additional file 1.** Supplemental Tables 1–3.

## Data Availability

The datasets supporting the conclusions of the current study are included within the article along with additional files. More datasets are available from the corresponding author upon reasonable request.

## Abbreviations

P-chem: Prophylactic chemotherapy
GTN: Gestational trophoblastic neoplasia
CHM: Complete hydatidiform mole
PHM: Partial hydatidiform mole
hCG: Human chorionic gonadotropin
FIGO: International Federation of Genecology and Obstetrics
NCCN: National Comprehensive Cancer Network
