# Supplementary material for: The effect of prophylactic chemotherapy on treatment outcome of postmolar gestational trophoblastic neoplasia
Source: BMC Womens Health. 2023 Jan 2;23:1. doi: 10.1186/s12905-022-02134-w (PMC9806869; doi:10.1186/s12905-022-02134-w)
Supplement: Supplementary file 1 — Additional file 1. Supplemental Tables 1–3. [file 12905_2022_2134_MOESM1_ESM.doc]

Supplemental Table 1. The Sociodemographic data of hydatidiform mole with subsequent low-risk GTN

| Variables ^a^ | control (n=72) | P-chem(n=24) | *P* value |
| --- | --- | --- | --- |
| **Age (years)** | 29(18-54) | 29(21-53) | 0.916 |
| **Gravidity** | 2(1-6) | 3(1-7) | 0.029 |
| **Parity** | 1(0-3) | 1(0-2) | 0.594 |
| **Gestational age(weeks)** | 9.5(6.3-25.1) | 9.3(4.7-21.0) | 0.647 |
| **Histology of HM** |  |  |  |
| Complete HM | 66 | 23 | 0.676 |
| Partial HM | 6 | 1 |  |
| **hCG before evacuation (IU/L)** | 225000(113140-225000) | 200000(1000-1278808) | 0.585 |
| **Diameter of theca lutein cyst** |  |  |  |
| ≤6 cm | 27 | 5 | 0.535 |
| >6 cm | 3 | 1 |  |
| Missing data | 42 | 18 |  |
| **Uterus greater than the gravid date** |  |  |  |
| No | 21 | 2 | 0.149 |
| Yes | 7 | 3 |  |
| Missing data | 44 | 19 |  |
| **History of HM in previous gestation** |  |  |  |
| No | 72 | 23 | 0.250 |
| Yes | 0 | 1 |  |

^a^Continuous variables are reported as medians(range), and categorical variables are reported as raw numbers.

Supplemental Table 2. Sociodemographic data of hydatidiform mole with subsequent high-risk GTN

| **Variables^a^** | control (n=9) | P-chem(n=3) | *P* value |
| --- | --- | --- | --- |
| **Age (years)** | 28(18-41) | 21(20-33) | 0.405 |
| **Gravidity** | 3(1-7) | 1(1-2) | 0.178 |
| **Parity** | 1(0-2) | 0(0-0) | 0.118 |
| **Gestational age(weeks)** | 8.6(6.7-12.9) | 13.6(9.9-17.3) | 0.078 |
| **Histology of HM** |  |  |  |
| Complete HM | 9 | 1 | 0.045 |
| Partial HM | 0 | 2 |  |
| **hCG before evacuation (IU/L)** | 200000(60000-225000) | 190512(125000-256024) | 0.550 |
| **History of HM in previous gestation** |  |  |  |
| No | 9 | 3 |  |
| Yes | 0 | 0 |  |
| **Diameter of theca lutein cyst** |  |  |  |
| Missing data | 9 | 3 |  |
| **Excessive uterine enlargement** |  |  |  |
| No | 1 | 0 |  |
| Yes | 0 | 1 |  |
| Missing data | 8 | 2 |  |

^a^Continuous variables are reported as medians(range), and categorical variables are reported as raw numbers.

Supplemental Table 3. Chemotherapy cycles of 22 low-risk GTN patients received MTX for P-chem

| **Variables^a^** | Group A (n=11) | Group B (n=11) | *P* value |
| --- | --- | --- | --- |
| **Chemotherapy cycles to hCG normalization** | 3(2-5) | 2(1-4) | 0.004 |
| **Total chemotherapy cycles** | 5(2-8) | 3(1-6) | 0.081 |

Group A: patients received MTX as first-line agent for GTN.

Group B: patients received alternative agent as first-line treatment for GTN.
